# Rubella metapopulation dynamics and importance of spatial coupling to the risk of congenital rubella syndrome in Peru

**DOI:** 10.1098/rsif.2010.0320

**Published:** 2010-07-21

**Authors:** C. J. E. Metcalf, C. V. Munayco, G. Chowell, B. T. Grenfell, O. N. Bjørnstad

**Affiliations:** 1 Department of Ecology and Evolutionary Biology, Eno Hall, Princeton University, Princeton, NJ 0854, USA; 2 Dirección General de Epidemiología, Lima, Peru; 3 Fogarty International Center, National Institutes of Health, Bethesda, MD 20892, USA; 4 Mathematical, Computational & Modeling Sciences Center, School of Human Evolution and Social Change, Arizona State University, Tempe, AZ 85287-2402, USA; 5 Center for Infectious Disease Dynamics, The Pennsylvania State University, 208 Mueller Lab, University Park, PA 16802, USA

**Keywords:** rubella, epidemiology, metapopulation, congenital rubella syndrome

## Abstract

Rubella is generally a mild childhood disease, but infection during early pregnancy may cause spontaneous abortion or congenital rubella syndrome (CRS), which may entail a variety of birth defects. Consequently, understanding the age-structured dynamics of this infection has considerable public health value. Vaccination short of the threshold for local elimination of transmission will increase the average age of infection. Accordingly, the classic concern for this infection is the potential for vaccination to increase incidence in individuals of childbearing age. A neglected aspect of rubella dynamics is how age incidence patterns may be moulded by the spatial dynamics inherent to epidemic metapopulations. Here, we use a uniquely detailed dataset from Peru to explore the implications of this for the burden of CRS. Our results show that the risk of CRS may be particularly severe in small remote regions, a prediction at odds with expectations in the endemic situation, and with implications for the outcome of vaccination. This outcome results directly from the metapopulation context: specifically, extinction–re-colonization dynamics are crucial because they allow for significant leakage of susceptible individuals into the older age classes during inter-epidemic periods with the potential to increase CRS risk by as much as fivefold.

## 1. Introduction

Rubella is primarily a relatively mild childhood disease (average age of infection approx. 7–12; [1]). However, when the disease is acquired during the first 16 weeks of pregnancy, the infection has severe consequences including foetal death or children born with congenital rubella syndrome (CRS), a condition associated with debilitating complications including hearing impairment, blindness and brain damage [2–4].

For strongly immunizing childhood infections, the average age of infection is expected to be relatively high if transmission is low, or where vaccination has been implemented. Generally, a higher average age of infection may be associated with an increased incidence in woman of childbearing age. How the magnitude of transmission and the degree of vaccination coverage affect the burden of CRS has been the focus of a range of modelling studies [5–9]. Recently, however, a new concern has been raised. The average age of infection may also be increased by dynamics characterized by episodic outbreaks in epidemic metapopulations [10]. This will also increase the number of cases in the risk group of women of childbearing age.

There is reason to anticipate episodic outbreaks for rubella: estimates of *R*_0_ for rubella are generally low—particularly relative to other childhood infections such as measles and pertussiss—typically between 3 and 8 [1,11,12], so that transient dynamics alone may lead to variable outbreak size across years [13]. The low *R*_0_ of this infection also enhances the probability that local chains of transmission will be broken because low *R*_0_ increases the critical community size (CCS), i.e. the population size above which rubella does not suffer stochastic extinction during recurrent post-epidemic troughs (e.g. [14–16]). The CCS for rubella has been estimated to be around 1 million in Peru [12] and Mexico [17]. This is a lot higher than the CCS of 300–550 k for a higher *R*_0_ infection such as measles [18].

In a spatial context, stochastic extinction and re-colonization dynamics resulting from a high CCS can potentially be a very important influence on age incidence curves, since such episodic dynamics may allow leakage of susceptible individuals into older age classes [10]. Consequently, characteristics of the metapopulation may shape where the burden of CRS is focused, and how this burden may change following routine vaccination. Here, we explore this question using a uniquely detailed dataset from Peru. We investigate the metapopulation effect by comparing expectations of the burden of CRS in the endemic situation with the empirical pattern observed across 175 provinces of Peru. In order to do this we first estimate the relative age-specific force of infection (FOI) across Peru, and infer from this the expected burden of CRS within the endemic Peruvian context. Second, we compare this with the indirectly observed burden as inferred from the proportion of rubella cases occurring in individuals aged more than 15 in each of the 175 provinces. Finally, combining rubella incidence and demographic data, we reconstruct the susceptible profiles for rubella and estimate seasonal variation in transmission rates and the degree of spatial isolation of each province. We find that the metapopulation effect results in an approximately fivefold increase in the burden of CRS in the isolated provinces.

## 2. Material and methods

### 2.1. Data

**Figure 1.**
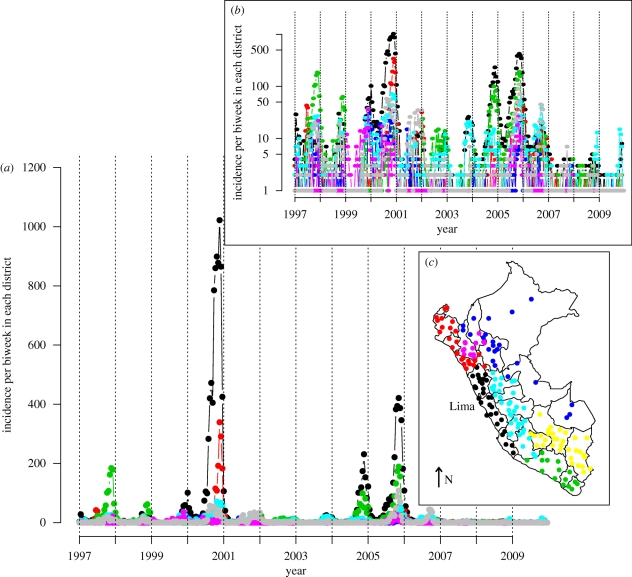
Rubella incidence in Peru. (*a*) Raw time series for seven geographical units corresponding to 175 provinces across 13 years, showing the Costa central (black line), Costa norte (red line), Costa sur (green line), Selva (blue line), Sierra central (turquoise line), Sierra norte (purple line) and Sierra sur (grey line); (*b*) log-transformed time series +1 for the same geographical units; (*c*) map of Peru showing locations of each of the geographical units, with colours corresponding to colours used in the time series; arrow indicates North.

**Figure 2.**
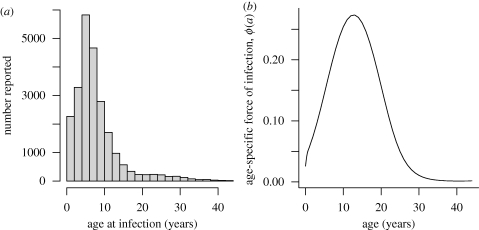
(*a*) Distribution of incidence over age taken from the entire country and (*b*) relative age-specific force of infection (FOI) over age, fitted with a smoothing spline with 5 d.f. Transmission is highest in school-age children, peaking around age 13.

The data from the Peruvian Health Ministry's Department of Epidemiology consist of weekly time series of reported rubella incidence from 1997 to 2009, stratified by province (figure 1) and by age (figure 2*a*). Case reporting in Peru followed the standard WHO guidelines [12] during this period. The dataset reports on a total of 24 116 cases. The country-wide average age of infection was 8 years, and, within any single week, the case numbers ranged from 0 to 772 reported cases (figure 1). Country-wide outbreaks, further, follow a predominantly annual pattern [12]. At a finer scale, however, rubella incidence is variable in space and time, reflecting heterogeneities inherent in the underlying epidemic metapopulation. The provinces vary in density from 12 people per km^2^ in the forested west regions of the country, to more than 172 people per km^2^ in the coastal region. The incidence reports were available for 175 of the provinces, ranging in population size from 7000 to more 6 million. Population size and birth rates were obtained from the database of the National Institute of Statistics and Informatics of Peru [19]. Routine vaccination and vaccination of individuals aged 15–19 was initiated in 2003–2005, but coverage during this period was low and estimated to be less than 4 per cent [12]. Enhanced routine vaccination of children was implemented in 2007; the coverage since ranges between 75 and 100 per cent.

### 2.2. Age-specific force of infection

Estimating the predicted burden of CRS in Peru-like settings in an endemic situation requires an estimate of the age-specific FOI, or the age-specific rate at which susceptible individuals become infected. We estimate the relative age-specific FOI, *φ*(*a*), using the catalytic framework [20]. The probability, *P*(*a*), of infection by age *a* is2.1
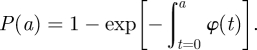
The probability of infection during childbearing age (between around age 15 and 45) is2.2
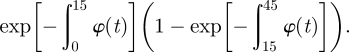
Because only a subset of cases are reported, the fraction of any local population that is never infected is not known; *P*(*a*) for individuals of very great ages will be less than 1 by an unknown fraction. This uncertainty prevents estimation of the absolute magnitude of the FOI. However, the relative age-specific FOI can be estimated by using the cumulative number of reported cases as the denominator [10]. We estimate the relative age-specific FOI using a cubic smoothing spline with 5 d.f. We fit the spline by maximizing a binomial likelihood using a quasi-Newton algorithm to find maximum-likelihood coefficients for each of the five basis functions of a cubic spline with 5 d.f. We invert the numerically evaluated Hessian to obtain a variance–covariance matrix for the spline coefficients. Re-sampling from this matrix allows us to erect error bounds on the age-specific FOI curve.

### 2.3. Fitting the time-series susceptible–infected–recovered model

If reporting rates are stable through time, and all individuals eventually succumb to infection, the numbers of susceptible individuals at time *t*, *S*_*t*_, in any given location will track births and infecteds, *I*_*t*_. Note that in this section on local dynamics we suppress the site-specific subscript for ease of notation. The pattern of susceptible individuals through time can then be reconstructed [21,22] by applying the balance equation2.3
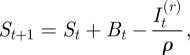
where *B*_*t*_ is the number of births (and may be discounted by vaccination where necessary), *ρ* is the reporting rate and 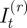 is the reported number of infected cases. Ignoring observational uncertainty, 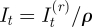, where *I*_*t*_ is the actual number of infected individuals at *t*. Rearranging equation (2.3) provides the relationship from which the reporting rate and the dynamics of the susceptible population can be inferred through susceptible reconstruction,2.4
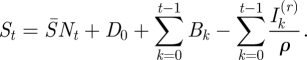


In equation (2.4), 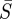 represents the average proportion of individuals that are susceptible, *N*_*t*_ represents the population size and *D*_0_ is the unknown deviation around the average at the time of the first observation in the time series. To estimate *ρ* and reconstruct a full time series of susceptible ‘deviations’, *D*_*t*_, that details how the numbers of susceptible individuals vary around the average number of susceptible individuals, we write2.5
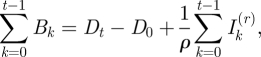
where 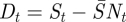. From this, *D*_*t*_ can be estimated as the residuals from the possibly locally varying regression of the cumulative number of births on the cumulative number of cases, and *ρ* can be estimated as the inverse slope of this regression [21–23]. Note that the average number of susceptible individuals cannot be directly estimated, as it is confounded with the intercept of this regression equation.

From this foundation, seasonal transmission rates can be estimated using time-series susceptible–infected–recovered (TSIR) methods [21,23]. The generation time (serial interval) of rubella (approximately the latent plus infectious period) is approximately 18 days [1,3], so we assumed that the time scale of the epidemic process was approximately two weeks, and aggregated the data accordingly. The number of infected individuals at time *t*, *I*_*t*+1_, depends stochastically on *I*_*t*_ and the number of susceptible individuals *S*_*t*_ with expectation *λ*_*t*_ = *β*_*s*_ *S*_*t*_ *I*_*t*_^*α*^, where *β*_*s*_ is the transmission rate in every biweek in any particular location and the exponent *α*, usually a little less than 1, captures heterogeneities in mixing not directly modelled by the seasonality [21,23] and the effects of discretization of the underlying continuous time process [24]. Then, taking logs on both side of this relationship, we can write2.6
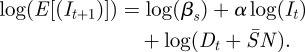
Given estimates of *I*_*t*_ and D_*t*_, regression techniques can be used to estimate *β*_*s*_ and *α* and marginal profile likelihoods can be used to estimate 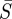 [22,23]. The transmission rate estimated in this way may reflect a broad range of processes that occur consistently over the course of a year. For childhood infections, low transmission usually mirrors periods of school vacation, indicating that the parameter captures mixing among school children [25]; for other infections, climatic variables such as absolute humidity (e.g. [26]) may be more important.

### 2.4. Connectivity

Estimates of under-reporting obtained via susceptible reconstruction and birth rates in every location provide the means to estimate the profile of susceptibility in each province, as described above. With this and biweekly transmission rates, a coupling parameter can be estimated for each province [27]. During fadeouts, in location *j*, the probability that no epidemic results following a spatial contact is 1/(1 + *β*_*s*_ *S*_*t,j*_), and, conversely, a new epidemic is sparked according to
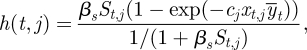
where *c*_*j*_ is a parameter that describes the coupling of community *j* to the regional metapopulation; *x*_*t,j*_ is the local proportion of susceptibility (*S*_*t,j*_/*N*,_*j*_,) and 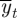 is the probability that a non-local individual is infectious (
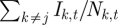
). Methods from survival analysis can be used to estimate *c*_*j*_ for every location [27].

Transmission in childhood infections in industrialized countries usually scales in a frequency-dependent fashion [28], since classroom size is typically relatively constant [23]. Therefore, it is convenient to consider the alternative parameterization of the TSIR where *λ_t_* = *β*′ *S*_*t*_ *I*_*t*_^*α*^/*N* where *β*′ = *β*′*N*. If transmission of rubella in Peru scales in a frequency-dependent fashion, the estimated magnitude of *β*_*s*_ from the TSIR relationship defined and fitted as described above (*λ*_*t*_ = *β*_*s*_ *S*_*t*_ *I*_*t*_^*α*^) should scale inversely with population size. A linear regression relating the log median value of *β*_*s*_ estimated for each of the seven regions (for which time series are shown in figure 1) with the log population size of that region has a slope very close to −1, in accordance with frequency-dependent scaling [23]. The relationship is not significant (*y* = 6.55–0.97*x*, *p* > 0.5, d.f. = 5, *r*^2^ = 0.25), but only seven regions were available. Since standard errors on regional estimates were considerable owing to low local incidence, we used the Peru-wide estimate of seasonality in transmission, and adjusted the median value for each location by the ratio between the local population size and the size of the entire population of Peru.

## 3. Results

### 3.1. Local dynamics

Only 0.3 per cent of observations occurred in ages greater than 45. Since the catalytic model is quite sensitive to rare cases in high age-classes, we discarded them, leaving 24 048 observations (figure 2*a*). The estimated pattern of relative FOI over age peaks in school children (around age 13), suggesting transmission predominantly in school children (figure 2*b*). The FOI then falls off relatively steeply. From this pattern over age, we can estimate the expected proportion of rubella cases in individuals aged greater than 15 as 0.04 in the endemic situation. Uncertainty bounds from re-sampling the variance–covariance matrix indicate a plausible range from 0.03 to 0.08. If we use the whole dataset (including age classes greater than 45) the predicted proportion increases to 0.07–0.08, depending on the number of degrees of freedom used for the spline. This estimate is, however, quite sensitive to the fact that the FOI is poorly estimated at high age-classes, a problem in all cross-sectional studies of age-specific FOI [29]. The same overall patterns are obtained if data are restricted to the period before the 2007–2009 vaccination campaign.

**Figure 3.**
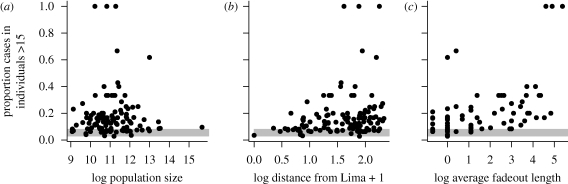
Observed proportion of cases in individuals aged greater than 15 (indicator of CRS burden) against (*a*) log population size (no significant correlation, *ρ* = −0.009, d.f. = 114, *p* > 0.5 for the full dataset; and *ρ* = 0.05, d.f. = 111, *p* > 0.5 for the analysis with the three points where the proportion is equal to 1 are removed); (*b*) log distance from Lima + 1 (significant positive correlation, *ρ* = 0.24, d.f. = 114, *p* < 0.01 for the full dataset; and *ρ* = 0.24, d.f. = 111, *p* < 0.01 for the analysis with the three points where the proportion is 1 are removed); and (*c*) log average fadeout length in biweeks (significant positive correlation, *ρ* = 0.55, d.f. = 114, *p* < 1e-9 for the full dataset; and *ρ* = 0.40, d.f. = 111, *p* < 1e-5 for the analysis with three points removed). Grey bars indicate the expected proportion of cases occurring in individuals greater than 15 years of age in the endemic situation based on the estimated relative FOI over age.

Figure 3 shows the proportion of incidence occurring in individuals older than 15 against population size, distance from Lima, and fadeout length (average number of consecutive zeros in the time series) (figure 3) and also shows the predicted range in the endemic setting (0.03–0.08; figure 2). Distance from Lima and fadeout length were both correlated with the proportion of cases aged less than 15. The effect of distance on this proportion was weak in smaller populations (*ρ* = 0.19, d.f. = 76, *p* < 0.1 for populations smaller than 700 000 versus *ρ* = 0.09, d.f. = 36, *p* < 0.01 for populations larger than 700 000); but accounting for this did not affect the overall relationships with population size and fadeout length.

**Figure 4.**
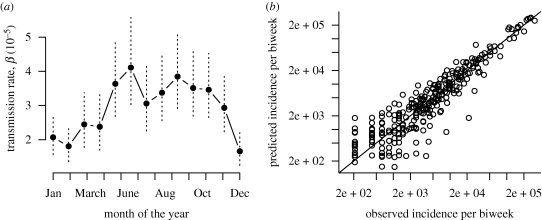
TSIR estimates of seasonal transmission rates showing (*a*) the pattern of transmission over the season, with standard errors shown as vertical dashed lines (the dip in transmission in July–August corresponds to the winter school holidays; and low transmission in December–March corresponds to the summer school vacation); and (*b*) the relationship between observed and expected numbers of total infected individuals in each biweek for the fitted model, where the observed is obtained by dividing through the reported numbers by the reporting rate (*ρ* = 0.005). The line indicates where *x* = *y*.

The estimated seasonal pattern of transmission reflects the timing of school holidays (figure 4*a*), with low transmission during the school summer vacations in Peru (21 December–1 March) and during the school winter vacations (24 July–3 August). This seasonality compares interestingly with patterns observed in childhood disease transmission in Europe: in both Peru and Europe the most pronounced trough in transmission occurs during the longest school vacation, but this occurs in July–August in Europe, versus December–March in Peru. The TSIR model provides a good fit to the short-term dynamics of the infection (figure 4*b*). For these estimates, we set *α* = 1, since fitted estimates of *α* were less than 0.9 (*α* = 0.87), a value that implies a substantial difference in transmission between epidemic troughs and peaks that is rather hard to interpret [23]. Repeating the analysis with *α* = 0.87 does not alter the qualitative conclusions of the paper; and the pattern of seasonality is identical for both analyses.

### 3.2. Spatial dynamics

**Figure 5.**
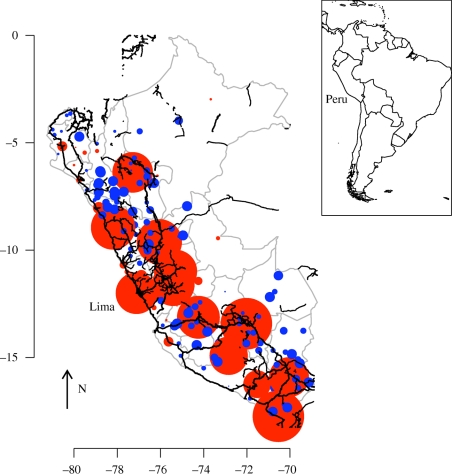
Positive (red) and negative (blue) residuals around the mean log-coupling parameter. The locations indicated by red points are more connected than average; these tend to be concentrated along major roads (thick black lines) or in road-dense areas (black lines). Blue points are generally either off the broader roads or on smaller roads.

**Figure 6.**
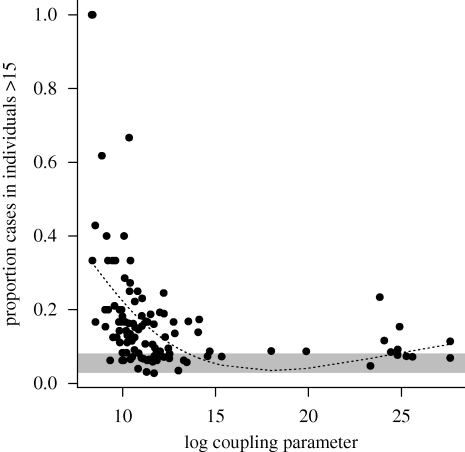
The observed proportion of cases in individuals aged greater than 15 (indicator of CRS burden) is significantly negatively correlated with the estimated-coupling parameter (*ρ* = −0.27, d.f. = 114, *p* < 0.005), as more weakly coupled locations will have longer fadeouts, and fadeout length is strongly linked to the average age of infection (figure 2). The grey bar indicates the expected proportion of cases occurring in individuals greater than 15 years of age in the endemic situation based on the estimated relative FOI over age. The dashed line shows a smooth spline with 3 d.f., used to estimate the average metapopulation burden for different degrees of coupling.

The estimated value of regional coupling significantly increased with population size and decreased with distance from Lima (log(*c*) = 5.98 + 0.67log(*N*) − 1.14log(*d* + 1), *p* < 0.05 for both coefficients). However, this relationship explained a small amount of the variance (*r*^2^ = 0.05), probably reflecting the complexity of the metapopulation structure in Peru. Highly coupled areas were concentrated along major roads (figure 5). A major new facet to the CRS story is that we found a significant negative correlation between the proportion of incidence occurring in individuals aged greater than 15 and the degree of coupling (figure 6).

## 4. Discussion

The major conclusion of our analysis is the importance of the metapopulation structure of Peru for rubella age–incidence curves and consequently for the spatial and temporal pattern of the risk of CRS. Heterogeneity in the degree of coupling of different locations across the country leads to highly variable rubella incidence (figure 1) and this affects the age profile of susceptibility (figure 6), the key variable in determining the burden of CRS. In locations that are weakly coupled to the metapopulation, regular local extinction resulting from the high CCS of rubella [12] will be followed by prolonged periods without re-introduction, which will allow susceptible individuals to leak into older age classes [10]. In agreement with this, a survey of sero-negativity in post-partum women in Peru identified considerable geographical structure, with much higher sero-negativity in the distant forest locations than in the costal region around Lima [30]. Additionally, CRS incidence in hospitals in, for example, Loreto, the weakly coupled province in the northeast of the country, are also high relative to central and coastal locations [31].

In an endemic situation, where transmission is invariant with population size, all else being equal, infant vaccination against rubella will reduce transmission globally, and the highest burden of CRS will be concentrated in large populations. By contrast, where rubella persists through metapopulation dynamics, with regular extinctions and re-introductions, *R*_0_ is not as critical as the accumulation of susceptible individuals that occurs following fadeouts. The highest burden of CRS will consequently be found in less connected locations. Previous analyses for measles in England and Wales might suggest that these would be smaller locations [27,32]; however, our analysis in Peru indicates that connectivity is more tightly determined by distance to Lima (figure 3), although small remote places remain particularly vulnerable given their higher potential for local extinction. This has public health ramifications, both in defining the burden of CRS and in considering allocation of effort in vaccination campaigns, reinforcing the importance of vaccination targeting women of childbearing age [33], but also emphasizing the spatial dimension of vaccine allocation. Locations where it is hardest to carry out vaccinations may also be the hardest to reach; and our results suggest that these may be precisely the locations with the highest cumulative CRS burden.

Our results also broaden our picture of the determinants of rubella dynamics more globally, highlighting for example the importance of transmission within schools, as evinced by both the peak in the relative age-specific FOI around 13 (figure 2, as compared with approx. 9 for England and Wales; [34]) and low transmission during school holidays (figure 4). The pattern of FOI over age estimated (figure 2) is overall similar to other estimates for rubella [29], although transmission is lower in later age classes; however, this qualitative difference might simply reflect poor estimation of the FOI over age at later age classes [29]. Evidence for a slope close to −1 characterizing the relationship between average transmission and population size also suggests that *R*_0_ is relatively invariant of population size. Since varying *R*_0_ is another potential determinant of regional variation in the burden of CRS, this provides valuable support for the suggested role of connectivity (figure 6), further reinforced by the link between fadeout length and proportion of cases occurring in individuals older than 15 years. This would not be expected if high average age was simply the result of lower transmission rates.

Our TSIR analysis assumes that reporting rates are homogeneous over space. If, as is reasonable to expect, in reality more remote locations have lower reporting rates, incidence in these locations will be underestimated, and susceptibility will be overestimated. The outcome will be higher estimates of connectivity for these remote locations, which is in the opposite direction of our expected effect, and therefore our conclusions are likely to be robust to this. More erratic reporting rates might also lead to biases; however, the link between connectivity and age of infection (a relatively independent indicator of this process) rather than simply extinction here provides further support that the pattern identified is a real one.

Given both variable presentation and distance from the initial infection, CRS is hard to detect [2,3], and direct observations on the burden of CRS are rare [35]. For much of the world, indirect measures based on the profile of susceptibility [36] constitute the best information we have. Our results suggest that this may underestimate the CRS burden considerably at a local scale. For example, at weakly coupled locations, with log coupling estimated at approximately 10, the predicted average proportion of cases in individuals greater than 15 is 0.22, i.e. a more than fivefold increase relative to the expected endemic estimate of 0.04. Although the dangers of inadequate vaccination implementation should be kept clearly in view [9], our results suggest that ignoring the metapopulation context may also result in an underestimate of the burden, and this might be an important consideration in the cost–benefit analyses of vaccine introduction in the context of increased interest in a global rubella control programme. However, it should also be considered that Peru may be a particularly extreme example of the metapopulation effect: a survey of rubella sero-negativity in women of childbearing age across a range of South American countries generally found no major differences in rural versus urban populations (Argentina, Brazil, Chile, Jamaica, Trinidad, Uruguay), but significant differences across Peru, with, for example, 40 per cent sero-negative in the northern forested part of the country, versus 20 per cent elsewhere [37]. Data of this form from a range of other countries allowing exploration of this issue would be of considerable value.

## References

[1] Anderson R. M. May R. M. 1991 Infectious diseases of humans. Oxford, UK: Oxford University Press

[2] Plotkin S. A. 2001 Rubella eradication. Vaccine 19, 3311–3319 10.1016/S0264-410X(01)00073-1 (doi:10.1016/S0264-410X(01)00073-1) 11348695

[3] Banatvala J. E. Brown D. W. G. 2004 Rubella. Lancet 363, 1127–1137 10.1016/S0140-6736(04)15897-2 (doi:10.1016/S0140-6736(04)15897-2) 15064032

[4] Best J. M. 2007 Rubella. Semin. Fetal Neonatal Med. 12, 182–192 10.1016/j.siny.2007.01.017 (doi:10.1016/j.siny.2007.01.017) 17337363

[5] Knox E. G. 1980 Strategy for rubella vaccination. Int. J. Epidemiol. 9, 13–23 10.1093/ije/9.1.13 (doi:10.1093/ije/9.1.13) 7419327

[6] Anderson R. M. May R. M. 1983 Vaccination against rubella and measles: qualitative investigations of different policies. J. Hyg. Camb. 90, 259–325 10.1017/S002217240002893X (doi:10.1017/S002217240002893X) 6833747 PMC2134248

[7] Anderson R. M. Grenfell B. T. 1986 Quantitative investigations of different vaccination policies for the control of congenital rubella syndrome (CRS) in the United Kingdom. J. Hyg. Camb. 96, 305–333 10.1017/S0022172400066079 (doi:10.1017/S0022172400066079) 3701044 PMC2129652

[8] Panagiotopoulos T. Antoniadou I. Valassi-Adam E. 1999 Increase in congenital rubella occurrence after immunisation in Greece: retrospective survey and systematic review. Br. Med. J. 319, 1462–1467 10582926 10.1136/bmj.319.7223.1462 PMC28289

[9] Vynnycky E. Gay N. J. Cutts F. T. 2003 The predicted impact of private sector MMR vaccination on the burden of congenital rubella syndrome. Vaccine 21, 2708–2719 10.1016/S0264-410X(03)00229-9 (doi:10.1016/S0264-410X(03)00229-9) 12798608

[10] Ferrari M. J. Djibo A. Grais R. F. Grenfell B. T. Bjørnstad O. N. 2010 Episodic outbreaks bias estimates of age specific force of infection: a corrected method using measles in Niamey, Niger as an example. Epidemiol. Infect. 138, 108–116 10.1017/S0950268809990173 (doi:10.1017/S0950268809990173) 19538818 PMC4520443

[11] Edmunds W. J. Gay N. J. Kretzschmar M. Wachmann H. 2000 The pre-vaccination epidemiology of measles, mumps and rubella in Europe: implications for modelling studies. Epidemiol. Infect. 125, 635–650 10.1017/S0950268800004672 (doi:10.1017/S0950268800004672) 11218214 PMC2869647

[12] Rios-Doria D. Chowell G. Munayco C. V. Whittembury A. Castillo-Chavez C. 2009 Spatial and temporal dynamics of rubella in Peru, 1997–2006: geographic patterns, age at infection and estimation of transmissibility. In Mathematical and statistical estimation approaches in epidemiology (ed. Chowell E. A.). Dordrecht, The Netherlands: Springer

[13] Bauch C. T. Earn D. J. D. 2003 Transients and attractors in epidemics. Proc. R. Soc. Lond. B 270, 1573–1578 10.1098/rspb.2003.2410 (doi:10.1098/rspb.2003.2410) PMC1691412 12908977

[14] Bartlett M. S. 1960 The critical community size for measles in the United States. J. R. Stat. Soc. Ser. A 123, 37–44

[15] Grenfell B. T. Harwood J. 1997 (Meta)population dynamics of infectious diseases. Trends Ecol. Evol. 12, 395–399 10.1016/S0169-5347(97)01174-9 (doi:10.1016/S0169-5347(97)01174-9) 21238122

[16] Nasell I. 2004 A new look at the critical community size for childhood infections. Theor. Popul. Biol. 67, 203–216 10.1016/j.tpb.2005.01.002 (doi:10.1016/j.tpb.2005.01.002) 15808337

[17] Metcalf C. J. E. Klepac P. Ferrari M. Bharti N. Bjørnstad O. N. Grenfell B. Submitted The epidemiology of rubella in Mexico: seasonality, stochasticity and regional variation. 10.1017/S0950268810002165 PMC3884048 20843389

[18] Bartlett M. S. 1957 Measles periodicity and community size. J. R. Stat. Soc. Ser. A Gen. 121, 48–70

[19] Admin 2010 Peru Instituto Nacional de Estadistica e Informatica. See http://www.inei.gob.pe/

[20] Griffiths D. A. 1974 A catalytic model of infection from measles. Appl. Stat. 23, 330–339 10.2307/2347126 (doi:10.2307/2347126)

[21] Finkenstadt B. Grenfell B. T. 2000 Time series modelling of childhood diseases: a dynamical systems approach. J. R. Stat. Soc. Ser. C 49, 187–205 10.1111/1467-9876.00187 (doi:10.1111/1467-9876.00187)

[22] Finkenstadt B. Bjørnstad O. N. Grenfell B. T. 2002 A stochastic model for extinction and recurrence of epidemics: estimation and inference for measles outbreaks. Biostatistics 3, 493–510 10.1093/biostatistics/3.4.493 (doi:10.1093/biostatistics/3.4.493) 12933594

[23] Bjørnstad O. N. Finkenstadt B. Grenfell B. T. 2002 Endemic and epidemic dynamics of measles: estimating epidemiological scaling with a time series SIR model. Ecol. Monogr. 72, 169–184

[24] Glass K. Xia Y. Grenfell B. T. 2003 Interpreting time-series analyses for continuous-time biological models-measles as a case study. J. Theor. Biol. 223, 19–25 10.1016/S0022-5193(03)00031-6 (doi:10.1016/S0022-5193(03)00031-6) 12782113

[25] Metcalf C. J. E. Bjørnstad O. N. Grenfell B. T. Andreasen V. 2009 Seasonality and comparative dynamics of six childhood infections in pre-vaccination Copenhagen. Proc. R. Soc. B 276, 4111–4118 10.1098/rspb.2009.1058 (doi:10.1098/rspb.2009.1058) PMC2821338 19740885

[26] Shaman J. Pitzer V. E. Viboud C. Grenfell B. T. Lipsitch M. 2010 Absolute humidity and the seasonal onset of influenza in the continental US. PLoS Biol. 8, e1000316 10.1371/journal.pbio.1000316 (doi:10.1371/journal.pbio.1000316) 20186267 PMC2826374

[27] Bjørnstad O. N. Grenfell B. 2008 Hazards, spatial transmission and timing of outbreaks in epidemic metapopulations. Environ. Ecol. Stat. 15, 265–277 10.1007/s10651-007-0059-3 (doi:10.1007/s10651-007-0059-3)

[28] McCallum H. Barlow N. Hone J. 2001 How should pathogen transmission be modelled. Trends Ecol. Evol. 16, 295–300 10.1016/S0169-5347(01)02144-9 (doi:10.1016/S0169-5347(01)02144-9) 11369107

[29] Shkedy Z. Aerts M. Molenberghs G. Beutels P. Van Damme P. 2006 Modelling age-dependent force of infection from prevalence data using fractional polynomials. Stat. Med. 25, 1577–1591 10.1002/sim.2291 (doi:10.1002/sim.2291) 16252265

[30] Suarez-Ognio L. Arianzen A. Ortiz A. Martinez C. Whittembury A. Cabezudo E. de Oliveira L. Siqueira M. M. Castillo-Solorzano C. 2007 A rubella serosurvey in postpartum women in three regions of Peru. Rev. Panam. Salud Publica 22, 110–117 17976277 10.1590/s1020-49892007000700005

[31] Blitchtein-Winick D. 2002 Sindrome de rubeola congenita en dicisiete hospitales del Peru, 1998-2000. An. Fac. Med. 63, 171–178

[32] Xia Y. Bjørnstad O. N. Grenfell B. T. 2004 Measles metapopulation dynamics: a gravity model for epidemiological coupling and dynamics. Am. Nat. 164, 267–281 15278849 10.1086/422341

[33] World Health Organization 2000 Rubella vaccine: WHO position paper. Wkly Epidemiol. Rec. 75, 161–172

[34] Grenfell B. T. Anderson R. M. 1985 The estimation of age-related rates of infection from case notifications and serological data. J. Hyg. Camb. 95, 419–436 10.1017/S0022172400062859 (doi:10.1017/S0022172400062859) 4067297 PMC2129533

[35] Lawn J. E. Reef S. Baffoe-Bonnie B. Adadevoh S. Caul E. O. Griffin G. E. 2000 Unseen blindness, unheard deafness, and unrecorded death and disability: congenital rubella in Kumasi, Ghana. Am. J. Public Health 90, 1555–1561 10.2105/AJPH.90.10.1555 (doi:10.2105/AJPH.90.10.1555) 11029988 PMC1446363

[36] Cutts F. T. Vynnycky E. 1999 Modelling the incidence of congenital rubella syndrome in developing countries. Int. J. Epidemiol. 28, 1176–1184 10.1093/ije/28.6.1176 (doi:10.1093/ije/28.6.1176) 10661666

[37] Dowdle W. R. 1970 WHO collaborative study on the sero-epidemiology of rubella in Caribbean and Middle and South American populations in 1968. Bull. World Health Organ. 42, 419–422 5310208 PMC2427532

